# Editorial: Case reports in general cardiovascular medicine: 2022

**DOI:** 10.3389/fcvm.2023.1271412

**Published:** 2023-08-23

**Authors:** Lijun Wang, Wensi Wan, Leonardo Roever, Pietro Enea Lazzerini, Junjie Xiao

**Affiliations:** ^1^ Cardiac Regeneration and Ageing Lab, Institute of Cardiovascular Sciences, Shanghai Engineering Research Center of Organ Repair, School of Life Science, Shanghai University, Shanghai, China; ^2^ Department of Clinical Research, Federal University of Uberlândia, Uberlândia, Brazil; ^3^ Gilbert and Rose-Marie Chagoury School of Medicine, Lebanese American University, Beirut, Lebanon; ^4^ Department of Medical Sciences, Surgery and Neurosciences, University of Siena, Siena, Italy

**Keywords:** case reports, prognosis, diagnosis, treatment, general cardiovascular medicine section, 2022

**Editorial on the Research Topic** Case reports in general cardiovascular medicine: 2022

In this 2022 Topic and Case Reports, an international collection of case reports is presented that aims to contribute to the advancement of understanding of personalized approaches to the prognosis, diagnosis, and treatment of cardiovascular disease. This editorial features the collection of Case reports published in Frontiers in Cardiovascular Medicine (General Cardiovascular Medicine Section) in 2022. In total 33 papers are published in this Research Topic. Case reports of the contributions in this special issue highlight unique cases that present with an unexpected diagnosis, treatment outcome, or clinical course. Hereby, reports on this issue can not only describe the cases' clinical presentation, diagnostic process, and treatment but also provide important experience and treatment strategies to manage patients with similar conditions. Following are the Case Reports that are published in the General Cardiovascular Medicine Section of the year 2022 (Table 1).

**Table 1:** Metrics (on July 7, 2023) of the articles published in case reports in general cardiovascular medicine: 2022.

Title	Authors	Views	Downloads
Amiodarone-Induced Multi-Systemic Toxicity Involving the Liver, Lungs, Thyroid, and Eyes: A Case Report	Hye-Su You, et al.	2,042	560
The Diagnostic Challenge of Eosinophilic Granulomatosis With Polyangiitis Presenting as Acute Eosinophilic Myocarditis: Case Report and Literature Review	Hiroyuki Yamamoto, et al.	3,044	541
Case Report: Intravascular Ultrasound-guided Intervention for Anastomosis Stenosis of the Left Main Coronary Artery Post-Cabrol Technique	Seok Oh, et al.	1,151	486
Aortic Coarctation Associated With Hypertrophic Cardiomyopathy in a Woman With Hypertension and Syncope: A Case Report With 8-Year Follow-Up	Hong Yang, et al.	1,094	444
Case report: Myocarditis following COVID-19 protein subunit vaccination	Yi-ming Dong, et al.	1,371	436
Case report: Aggressive progression of acute heart failure due to juvenile tuberculosis-associated Takayasu arteritis with aortic stenosis and thrombosis	Wenjie Xuan, et al.	496	430
Case report: Changes in the levels of stress hormones during Takotsubo syndrome	Pablo Ruiz, et al.	569	420
Inverted Takotsubo Syndrome With HELLP Syndrome: A Case Report	Paul Gabarre, et al.	1,162	419
Decisive diagnostic clue for infectious abdominal aortic aneurysm caused by Arthrobacter russicus in a diabetic elderly woman with renal dysfunction: A case report and literature review	Hiroyuki Yamamoto, et al.	1,838	401
Disrupting arrhythmia in a professional male wrestler athlete after rapid weight loss and high-intensity training—Case report	Aleksandra Milovančev, et al.	574	335
Myocardial infarction with non-obstructive coronary arteries in a patient double-seropositive for anti-glomerular basement membrane and anti-neutrophil cytoplasmic antibodies: A case report	Marcell Krall, et al.	1,121	312
Case Report: Danon Disease: Six Family Members and Literature Review	Yuanyuan Wang, et al.	957	310
Case report: Pulmonary arterial hypertension in ENG-related hereditary hemorrhagic telangiectasia	Dong Liu, et al.	803	283
Case Report: An Unusual Case of Pheochromocytoma	Ying Liao, et al.	881	276
Case Report: Family Curse: An SCN5A Mutation, c.611C>A, p.A204E Associated With a Family History of Dilated Cardiomyopathy and Arrhythmia	Wen Huang, et al.	1,252	270
Case report: From monkeypox pharyngitis to myopericarditis and atypical skin lesions	María Ascensión Sanromán Guerrero, et al.	639	266
Case report: Conquer a complex variant: Coronary-pulmonary artery fistulas, atrial septal defect and bicuspid pulmonary valve, under beating heart surgery	Ting Zhou, et al.	530	262
Case report: Coronavirus Disease 2019 (COVID-19) modified RNA vaccination-induced Adult-Onset Still's Disease with fulminant myocarditis as initial presentation	Mi Zhou, et al.	960	254
Case report: Sudden cardiorespiratory collapse in a healthy male after coronavirus disease 2019 vaccination at a vaccination center	Cze Ci Chan, et al.	1,118	247
Multisystem immune-related adverse events due to toripalimab: Two cases-based review	Yanran Chen, et al.	486	232
A case report and review of literature: Tuberculous pericarditis with pericardial effusion as the only clinical manifestation	Shipeng Wang, et al.	639	218
Case report: A novel surgical technique for rapid valve-in-ring implantation into the native aortic annulus during left ventricular assist device implantation	Yuriy Pya, et al.	512	215
Case report: Severe dry cough associated with superior vena cava syndrome—Caused recurrent chylothorax	Ziming Wan, et al.	760	212
Multimodal Imaging for Total Anomalous Systemic Venous Drainage Diagnosis and Preoperative Planning: A Case Report and Literature Review	Mingyan Ding, et al.	675	191
Massive pleural effusion following high-power and short-duration radiofrequency ablation for treatment of atrial fibrillation: A case report and review of the literature	Miaomiao He, et al.	1,012	179
Case report: Delayed cardiac rupture with congenital absence of pericardium after blunt trauma	Tuo Shen, et al.	333	164
Case report: Reducing the duration of positive-pressure ventilation for large mediastinal masses	Zaili Zhang, et al.	329	146
Case report: Metastatic myxoid liposarcoma arising from the right atrium extends as cardiac tamponade—A rare case of atrial oncology	Muralidharan Thoddi Ramamurthy, et al.	897	144
Right atrial cardiac lipoma with distinctive imaging characteristics. A rare case report and literature review	Qiqing Chen, et al.	492	142
Successful simultaneous stenting of a pulmonary artery and vein in pulmonary vascular stenosis due to silicosis. Case report and literature review	M. Westhoff, et al.	489	101
An adult female patient with single atrium and single ventricle undergoing appendectomy: A case report	Yu Wu, et al.	496	90
Acute myocardial infarction after inactivated COVID-19 vaccination: a case report and literature review	Weimei Ou, et al.	739	81
Case report: Aortoesophageal fistula—an extremely rare but life-threatening cardiovascular cause of hematemesis	Alexis Ching Wong, et al.	542	70

The order of appearance according to the number of downloads (on July 7, 2023).

*–Amiodarone-Induced Multi-Systemic Toxicity Involving the Liver, Lungs, Thyroid, and Eyes: A Case Report* (You et al.).

Amiodarone is a widely used anti-arrhythmia drug despite its side effects on various organs. However, cases of simultaneous toxicity of different organs in one patient have been rarely reported. This report describes an original case that multi-systemic amiodarone organ toxicity occurred in high-risk patients. This study provides important information for the diagnosis and treatment of amiodarone toxicity that could be useful for both physicians and researchers, as well as guideline makers in amiodarone administration.

*–The Diagnostic Challenge of Eosinophilic Granulomatosis With Polyangiitis Presenting as Acute Eosinophilic Myocarditis: Case Report and Literature Review* (Yamamoto et al.).

Eosinophilic granulomatosis with polyangiitis (EGPA) is a rare disease that is typically associated with infection and drug factors. It commonly affects multiple organs in the body and manifests with various discomforting symptoms. EGPA-associated eosinophilic myocarditis is rare but can lead to death if left untreated. This report describes a case with this rare disease cured by timely treatment with systemic and oral corticosteroids. The description in this report provides critical clinical features and therapy strategies that can be useful for physicians to identify and treatment of this disease.

*–Case Report: Intravascular Ultrasound-guided Intervention for Anastomosis Stenosis of the Left Main Coronary Artery Post-Cabrol Technique* (Oh et al.).

This study reports a case of acute myocardial infarction that is successfully treated with intravascular ultrasound (IVUS)-guided percutaneous coronary intervention (PCI). This is the first report that combined PVCI with virtual IVUS. The observations in this paper provide important information for physicians about how to evaluate plaque composition and conduct PCI in patients with chronic inflammatory diseases like Behçet's disease.

*–Aortic Coarctation Associated With Hypertrophic Cardiomyopathy in a Woman With Hypertension and Syncope: A Case Report With 8-Year Follow-Up* (Yang et al.).

Coarctation of the aorta (CoA) and hypertrophic cardiomyopathy (HCM) are two irrelevant cardiovascular diseases (1). This report describes a diagnosis, management, and 8-year follow-up in a patient with the co-existence of CoA and HCM. This is the first case that reports the concurrent presence of CoA and HCM in one patient. This report suggests that, albeit with low probability, CoA and HCM may co-exist in one patient, physicians should keep an eye on CoA in young hypertensive patients.

*–Case report: Myocarditis following COVID-19 protein subunit vaccination* (Dong et al.).

This study reports a case of myocarditis following ZF2001 vaccination in a female patient. This report describes the patient's clinical symptoms and immunohistochemical characteristics of heart sections. Though some patients with myocarditis following COVID-19 vaccination were vaccinated types of vaccines have been reported. Limited knowledge has been known about the association between vaccination and myocarditis in subunit vaccines against COVID-19. The report here suggests that physicians should consider the potential link between vaccination and myocarditis in clinical diagnosis. Also, investigating the specific mechanism underlying myocarditis after vaccination should be significant to guild the development of a new generation of subunit vaccines.

*–Case report: Aggressive progression of acute heart failure due to juvenile tuberculosis-associated Takayasu arteritis with aortic stenosis and thrombosis* (Xuan et al.).

Takayasu arteritis (TA) is a chronic granulomatous vasculitis of unknown pathophysiological mechanisms. The report describes a case of tuberculosis-associated TA who fail to survive after surgery. In this paper, the authors share their clinical experience in the diagnosis of TA and what they could learn from the case. The information provides in this paper can offer important knowledge for physicians to timely diagnose TA at an early stage with few typical features.

*–Case report: Changes in the levels of stress hormones during Takotsubo syndrome* (Ruiz et al.). *Inverted Takotsubo Syndrome With HELLP Syndrome: A Case Report* (Gabarre et al.).

Takotsubo syndrome is a sudden, transient, acute episode of left ventricular dysfunction, usually triggered by physical or emotional stress (2). These two reports describe an unusual case of Takotsubo syndrome which is accompanied by HELLP syndrome in a postpartum woman after spontaneous vaginal delivery. These studies provide indicative information for physicians that, in addition to non-invasive cardiac imaging, detecting circulating cortisol and cardiac biomarkers can be a useful complement strategy to diagnose Takotsubo syndrome. Further efforts are also warranted for researchers to identify more cardiac biomarkers in assisting the diagnosis of this disease.

*–Decisive diagnostic clue for infectious abdominal aortic aneurysm caused by Arthrobacter russicus in a diabetic elderly woman with renal dysfunction: A case report and literature review* (Yamamoto et al.).

Infectious aortic aneurysm (IAA) is a rare but serious infectious inflammatory disease of the aortic wall with high mortality. This report describes a case of IAA caused by *Arthrobacter russicus* that is successfully treated with a combination of surgery and long-term antimicrobial therapy. *Arthrobacter russicus* is identified as a potential causative microorganism by using 16S rRNA sequencing. This paper indicates that a molecular diagnosis is important for identifying the causative microorganism, in particular in cases of Gram staining and tissue culture-negative IAA.

*–Disrupting arrhythmia in a professional male wrestler athlete after rapid weight loss and high-intensity training—Case report* (Milovančev et al.).

It is well-known that physical exercise can induce physiological cardiac adaptions and benefit cardiovascular health (3, 4). However, in some cases, physiological heart adaptations may lead to increased susceptibility to arrhythmia in athletes. In this paper, the authors report a case of a wrestler athlete who developed disrupting arrhythmia during rapid weight loss and high-intensity training. This report provides useful information for athletes' physicians that precise intensive training prescriptions for individuals should be given by cardiovascular screening.

*–Myocardial infarction with non-obstructive coronary arteries in a patient double-seropositive for anti-glomerular basement membrane and anti-neutrophil cytoplasmic antibodies: A case report* (Krall et al.).

Anti-neutrophil cytoplasm antibody (ANCA)-associated vasculitides (AAV) and anti-glomerular basement membrane (GBM) disease are rare diseases with high mortality. This report describes a myocardial infarction with non-obstructive coronary arteries (MINOCA) patient with double seropositive for anti-ANCA and anti-GBM antibodies. Limited cases have been reported of anti-GBM disease patients with cardiovascular complications. These observations provide important information for physicians that endothelial injury or systemic inflammation patients with double-positive disease might be at higher risk to develop MINOCA. Further in-depth investigations to understand the clear causality and mechanism are required in the future.

*–Case Report: Danon Disease: Six Family Members and Literature Review* (Wang et al.).

Danon disease is a rare X-linked dominant genetic disorder characterized clinically by hypertrophic cardiomyopathy, skeletal muscle weakness, and intellectual disability (5). This report describes a case of Danon disease and his family members and reviews other relevant literature to discuss the clinical symptoms and possible strategies to treat Danon disease. This study offers critical information for the diagnosis and treatment of this disease.

*–Case report: Pulmonary arterial hypertension in ENG-related hereditary hemorrhagic telangiectasia* (Liu et al.).

The etiology of pulmonary arterial hypertension (PAH) can stem from various factors and is often multifactorial. It is crucial to implement standardized diagnostic procedures, investigate potential causes, and identify risk factors early on to enhance patient prognosis. In this paper, the authors report a case of hereditary hemorrhagic telangiectasia (HHT) combined with PAH. The clinical presentation, diagnostic process, and treatment of this rare case report provide useful information for physicians to diagnose HHT combined PAH patients. Further studies to better the pathogenesis of PAH in HHT should be of contribution to the timely and accurate diagnosis of this disease.

*–Case Report: An Unusual Case of Pheochromocytoma* (Liao et al.).

This report describes a female patient with acquired long QT syndrome, which is a rare complication of pheochromocytoma. Limited to the rarity and nonspecific clinical manifestations, diagnosis of pheochromocytoma is difficult. This study provides a successful diagnosis procedure of pheochromocytoma in this reported case and can offer important points for physicians to identify rare causes of common symptoms.

*–Case Report: Family Curse: An SCN5A Mutation, c.611C>A, p.A204E Associated With a Family History of Dilated Cardiomyopathy and Arrhythmia* (Huang et al.).

This study reports that *SCN5A* mutation with various dilated cardiomyopathy (DCM) associated gene mutations result in multiple phenotypes in a 3-generation family. By analyzing clinical data and genetic testing, this report suggests that a combination of *SCN5A* and PRKAG2 mutation can lead to DCM plus multifocal ectopic Purkinje-related premature contractions and PRKAG2 syndrome. This study discusses the possible causality between gene variants and observed clinical phenotype and indicates that gene diagnosis can offer early intervention to help physicians to improve diagnoses of DCM.

*–Case report: From monkeypox pharyngitis to myopericarditis and atypical skin lesions* (Guerrero et al.).

In this report, the authors describe a case of myopericarditis which is caused by human monkeypox, which is an endemic disease, infection followed by the onset of atypical skin lesions. This paper presents the clinical presentation, and diagnostic experience of this patient and provides antiviral treatment recommendations for physicians.

*–Case report: Conquer a complex variant: Coronary-pulmonary artery fistulas, atrial septal defect and bicuspid pulmonary valve, under beating heart surgery* (Zhon et al.).

This report describes a rare patient with two coronary artery to pulmonary artery fistula (CPAF) from two branches of the left anterior descending coronary to the main pulmonary artery, coexisting with a bicuspid pulmonary valve and atrial septal defect and treated by on-pump beating-heart surgery (OPBHS). This study discusses the potential origin and reason of this case as well as the clinical advantages of OPBHS. This report is a very complex case that has not yet been previously reported in the literature. This study offers important information for surgeries to treat CPAFs by OPBHS for patients with low surgical risk.

*–Case report: Coronavirus Disease 2019 (COVID-19) modified RNA vaccination-induced Adult-Onset Still’s Disease with fulminant myocarditis as initial presentation* (Zhou et al.).

Several types of vaccines against the coronavirus disease 2019 (COVID-19). In this paper, the authors report a case of an elderly female who is diagnosed with post-vaccination about Adult-Onset Still's Disease after receiving a modified mRNA vaccine (BNT162b2). Adult-Onset Still's Disease is a rare autoinflammatory disease and potentially life-threatening. The report here describes the clinical presentation and diagnosis of this rare Adult-Onset Still's Disease following mRNA vaccines can provide important knowledge for physicians about this disease.

*–Case report: Sudden cardiorespiratory collapse in a healthy male after coronavirus disease 2019 vaccination at a vaccination center* (Chan et al.).

This paper reports a case of sudden cardiorespiratory collapse in a healthy young male after receiving the Oxford-AstraZeneca (ChAdOx1 nCoV-19) COVID-19 vaccination. This report addresses the importance of careful medical attention by healthcare providers to avoid possible severe adverse events following COVID-19 vaccination which could be certainly useful for healthcare policymakers, and physicians.

*–Multisystem immune-related adverse events due to toripalimab: Two cases-based review* (Chen et al.).

Immune checkpoint inhibitors (ICIs) significantly prolong survival in patients with advanced cancer. However, immune-mediated adverse events (irAEs) caused by ICIs pose a major threat to patients' lives. In this paper, the authors report 2 patients with advanced tumors suffering from severe multisystem irAEs following treated with toripalimab (anti-PD-1). This report also reviews the Case reports regarding multisystem irAEs based on myocarditis induced by PD-1 inhibitors. Early detection and diagnosis of multisystem inflammation are critical for the management of patients treated with ICIs. This study can provide important experience and critical information for physicians to manage patients undergoing ICIs treatment.

*–A case report and review of literature: Tuberculous pericarditis with pericardial effusion as the only clinical manifestation* (Wang et al.).

Tuberculosis (TB) remains a serious public health problem worldwide. In this paper, the authors report a case of a patient with Tuberculous pericarditis (TBP) who is admitted to the hospital with massive pericardial effusion. This patient is examined as negative with all routine laboratory tests for the pericardial effusion of unknown origin. This report provides experience and information for physicians to diagnose and treat percardial effuion patients, in particular to those demonstrated as non-TB causes negative.

*–Case report: A novel surgical technique for rapid valve-in-ring implantation into the native aortic annulus during left ventricular assist device implantation* (Pya et al.).

Left ventricular assist device (LVAD) implantation is an excellent option for treating end-stage heart failure patients (6). This report describes a novel and feasible surgical technique for performing transcatheter aortic valve replacement in valve-in-ring along with LVAD implantation for the treatment of a male patient suffering from refractory heart failure due to dilated cardiomyopathy and pure aortic insufficiency in need of a new aortic bioprosthesis. This study reports a novel surgical technique that can share treatment experience for cardiologists in aortic valve repair.

*–Case report: Severe dry cough associated with superior vena cava syndrome—Caused recurrent chylothorax* (Wan).

This case reports a patient with uremia who suffered from severe dry cough. This patient is finally diagnosed with superior vena cava and treated by percutaneous balloon dilatation. Chronic coughs, which may be caused by many diseases, are very common syndrome in the respiratory department outpatient clinic. This paper reports a severe dry cough case caused by superior vena cave occlusion-induced recurrent chylothorax can provide information for physicians to diagnose recurring severe cough patients.

*–Multimodal Imaging for Total Anomalous Systemic Venous Drainage Diagnosis and Preoperative Planning: A Case Report and Literature Review* (Ding et al.).

Total anomalous systemic venous drainage (TASVD) is a rare congenital heart disease (7). In this case report, the authors report a 40-year-old male patient with TASVD. As a rare heart disease, limited information can be obtained from a literature review about the description of TASVD except Case reports. Diagnosing TASVD would be a difficult task in the medical field, primarily because of the variations observed in individuals and the absence of specific laboratory tests. In general, echocardiography is the first-line examination for TASVD diagnosis. This report provides information for physicians that multimodal imaging (Transthoracic, transesophageal, 3D echocardiography, contrast echocardiography, and computed tomography angiography) can be performed to verify the diagnosis of TASVD.

*–Massive pleural effusion following high-power and short-duration radiofrequency ablation for treatment of atrial fibrillation: A case report and review of the literature* (He et al.).

Radiofrequency catheter ablation (RFCA) is a non-pharmacological treatment for drug-refractory atrial fibrillations. Postpericardial injury syndrome (PPIS) is defined as pericarditis or pericardial effusion during myocardial infarction or cardiac intervention. It is widely recognized as a common complication of cardiac surgery, with an incidence ranging from 3% to 30%. Different from previously reported PPIS which is most characterized by pericardial effusion, this report describes a case of PPIS that presents pleural effusion as a main feature after the operation. This case report also systematically reviews the clinical characteristics of PPIS. The study in this paper provides useful reference information for physicians to timely diagnose a patient with massive pleural effusion alone following RFCA.

*–Case report: Delayed cardiac rupture with congenital absence of pericardium after blunt trauma* (Shen et al.).

As a rare disease with no specific symptoms and signs, the clinical diagnosis of congenital absence of pericardium (CAP) is very difficult. In this paper, the authors report a case of a patient with CAP and present some atypical features which are different from previously reported literature. The clinical presentation and diagnostic process of this patient should be useful to physicians in the diagnosis and treatment of CAP.

*–Case report: Reducing the duration of positive-pressure ventilation for large mediastinal masses* (Zhang et al.).

This report describes two high-risk patients with large mediastinal tumors (MMs) during general anesthesia undergoing tumorectomy by thoracotomy. The perioperative management of patients with large MMs is challenging. This study suggests that patients with large MM should receive positive-pressure ventilation following muscle relaxants given, but that cardiothoracic surgeons should immediately transect the sternum to relieve compression. This study provides important information and experiences for physicians to manage patients with large MMs.

*–Case report: Metastatic myxoid liposarcoma arising from the right atrium extends as cardiac tamponade—A rare case of atrial oncology* (Ramamurthy et al.). *Right atrial cardiac lipoma with distinctive imaging characteristics. A rare case report and literature review* (Chen et al.).

Cardiac lipomas is rare primary cardiac tumors, many of them do not exhibit obvious features and are difficult to identify. These two reports describe patients presenting a lipomas in the right atrium. In these papers, the authors share their experimence in the diagnosis and treatment of cardiac lipomas can provide useful information for physicians.

*–Successful simultaneous stenting of a pulmonary artery and vein in pulmonary vascular stenosis due to silicosis. Case report and literature review* (Westhoff et al.).

This report describes a successful stenting of a pulmonary artery and vein in a silicosis-caused pulmonary vascular stenosis patient. In this paper, the authors provide interventional procedure recommendations for this pulmonary vascular stenosis for physicians *via* clinical symptom presentation and literature review.

*–An adult female patient with single atrium and single ventricle undergoing appendectomy: A case report* (Wu et al.).

This paper reports an adult female patient with a single atrium and single ventricle undergoing appendectomy. Single atrium and single ventricle is a rare heart disease with very few patients surviving into adulthood. This report describes their associated surgical findings and can share useful experiences for physicians to diagnose patients with similar conditions.

*–Acute myocardial infarction after inactivated COVID-19 vaccination: a case report and literature review* (Ou et al.).

Many types of vaccines have been developed to restrain the pandemic of COVID-19. This study reports an acute myocardial infarction case following post-COVID-19 vaccination and discusses the potential pathogenesis mechanisms. This study provides clinical experience and recommendations for physicians to manage patients with similar conditions.

*–Case report: Aortoesophageal fistula—an extremely rare but life-threatening cardiovascular cause of hematemesis* (Wong et al.).

In this paper, the authors report a hematemesis patient with underlying diagnosed esophageal cancer who presents typical aortoesophageal fistula (AEF) clinical features. This report highlights the clinical features and diagnosis process of this extremely rare but life-threatening disease. This paper also provides recommendations for physicians to timely diagnose AEF.

In conclusion, the high-quality clinical case contributions presented in this Research Topic have significantly boosted knowledge, diagnosis, and treatment of cardiovascular disease in complex cases.
